# A novel prediction model of pancreatic fistula after pancreaticoduodenectomy using only preoperative markers

**DOI:** 10.1186/s12893-023-02213-1

**Published:** 2023-10-12

**Authors:** Hiroto Matsui, Yoshitaro Shindo, Daisaku Yamada, Hiroyuki Ogihara, Yukio Tokumitsu, Masao Nakajima, Michihisa Iida, Nobuaki Suzuki, Shigeru Takeda, Yuki Nakagami, Shogo Kobayashi, Hidetoshi Eguchi, Tatsuya Ioka, Yoshihiko Hamamoto, Hiroaki Nagano

**Affiliations:** 1 Department of Gastroenterological, Breast and Endocrine Surgery, Yamaguchi University Graduate School of Medicine, 1-1-1 Minami-Kogushi, Ube, Yamaguchi, Ube, 755-8505 Yamaguchi Japan; 2 Department of Gastroenterological Surgery, Graduate School of Medicine, Osaka University, Osaka, Japan; 3 Department of Computer Science and Electronic Engineering, National Institute of Technology, Tokuyama College, Shunan, Yamaguchi Japan; 4 Department of Data Science, Graduate School of Economics, Shimonoseki City University, Shimonoseki, Yamaguchi Japan; 5 Oncology Center, Yamaguchi University Hospital, Ube, Yamaguchi Japan; 6 Division of Electrical, Electronic and Information Engineering, Graduate School of Sciences and Technology for Innovation, Yamaguchi University, Ube, Yamaguchi Japan

**Keywords:** Postoperative pancreatic fistula, Pancreaticoduodenectomy, Discrete Bayes classifier, Prediction model

## Abstract

**Background:**

Since clinically relevant postoperative pancreatic fistula (CR-POPF) can cause intra-abdominal hemorrhage and abscesses, leading to surgery-related deaths after pancreaticoduodenectomy (PD), its preoperative prediction is important to develop strategies for surgical procedures and perioperative management. This study aimed to establish a novel prediction model for CR-POPF using preoperative markers.

**Methods:**

On a training set of 180 patients who underwent PD at the Yamaguchi University Hospital, a combination of CR-POPF predictors were explored using the leave-one-out method with a unique discrete Bayes classifier. This predictive model was confirmed using a validation set of 366 patients who underwent PD at the Osaka University Hospital.

**Results:**

In the training set, CR-POPF occurred in 60 (33%)　of 180 patients and 130 (36%)　of 366 patients in the validation set using selected markers. In patients with pancreatic ductal adenocarcinoma (PDAC), the main pancreatic duct (MPD) index showed the highest prognostic performance and could differentiate CR-POPF with 87% sensitivity and 81% specificity among 84 patients in the training set. In the validation set, the sensitivity and specificity of the MPD index-based model for 130 PDAC samples were 93% and 87%, respectively. In patients with non-PDAC, the MPD index/body mass index (BMI) combination showed the highest prognostic performance and could differentiate CR-POPF with 84% sensitivity and 57% specificity among 96 patients in the training set. In the validation set, the sensitivity and specificity of the MPD index/BMI-based model for 236 non-PDAC samples were 85% and 53%, respectively.

**Conclusion:**

We developed a novel prediction model for pancreatic fistulas after PD using only preoperative markers. The MPD index and MPD index/BMI combination will be useful for CR-POPF assessment in PDAC and non-PDAC samples, respectively.

**Supplementary Information:**

The online version contains supplementary material available at 10.1186/s12893-023-02213-1.

## Background

Pancreaticoduodenectomy (PD) is one of the most difficult surgical procedures. In recent years, the post-PD mortality rate at high-volume centers has decreased to < 2% owing to improvements in surgical techniques and perioperative management [1, 2]. However, post-PD morbidity rates remain high (16–50%) [3–5]. Among the most important post-PD complications, postoperative pancreatic fistula (POPF) can cause intra-abdominal hemorrhage and abscesses, leading to surgery-related deaths. Post-PD pancreatic fistula occurred in 15–45% of patients, according to the 2016 edition of the International Study Group of Pancreatic Surgery (ISGPS), and was associated with a mortality rate of up to 9% [6].

According to the ISGPS diagnostic criteria, POPF is classified as biochemical leak (without adverse clinical consequences) or clinically relevant POPF (CR-POPF), which is severe and requires various treatments. In recent years, proactive, risk-based management of pancreatic anastomosis has been advocated before serious complications occur with CR-POPF [7, 8]. Therefore, it is important to preoperatively predict CR-POPF risk, which is difficult with a single factor because POPF is associated with a number of confounders, including disease-related factors (such as pancreatic texture) and patient-related factors (such as obesity) [9, 10].

Therefore, in recent years, prediction models using multiple markers have been developed to predict POPF. However, most of them consist of intraoperative or postoperative markers, and few have preoperative predictive utility [11, 12]. This study aimed to predict CR-POPF with a high degree of accuracy by focusing solely on preoperative clinicoradiological data. We attempted to prevent missing CR-POPF. We extracted predictive markers of CR-POPF from previous literature and differentiated CR-POPF using a unique classifier [13].

## Methods

### Patients

For the training analysis, patients who underwent PD at the Department of Gastroenterological, Breast and Endocrine Surgery, Yamaguchi University Graduate School of Medicine, were selected. A total of 188 patients underwent PD between January 2010 and December 2020. Patients were excluded from the analysis if they met any of the following criteria: hepatopancreatoduodenectomy (n = 2) and two-stage pancreatojejunostomy [14] (n = 6). Ultimately, 180 patients were enrolled in the training set.

For the validation analysis, 371 patients, who underwent PD at the Department of Gastroenterological Surgery, Osaka University Graduate School of Medicine, from January 2011 to December 2020, were selected. Inclusion and exclusion criteria for study participation were similar to those for the training set. Patients were excluded from the analysis if they met any of the following criteria: hepatopancreatoduodenectomy (n = 2), PD for remnant pancreatic lesions (n = 2), and PD concomitant with colectomy for colon cancer (n = 1). Finally, 366 patients were enrolled in the validation set.

The collected data were statistically analyzed and assessed at Yamaguchi University. Approval from the institutional review board was obtained from each institution (IRB number: H2021-163). The study was conducted in accordance with Good Clinical Practice guidelines and the Declaration of Helsinki.

### Assessment of clinical and radiological findings

Each patient was evaluated using routine preoperative computed tomography (CT) or magnetic resonance imaging images and clinical data, including preoperative characteristics, postoperative complications, and histopathological diagnosis, from a prospectively maintained computer database. The collected data included patient age, sex, height, weight, body mass index (BMI),　presence/absence of diabetes, serum albumin level, total peripheral blood lymphocyte count, histopathological diagnosis, main pancreatic duct (MPD) size, stump thickness, stump width, preoperative biliary drainage (yes/no), and preoperative therapy. The prognostic nutritional index (PNI) [15] and controlling nutritional status (CONUT) score [16], indicators of nutritional status, were assessed in accordance with the following equation, as described previously.

Preoperative radiological markers included visceral fat area (VFA) [17], parenchymal thickness, MPD size, MPD index [18], presence of sarcopenia, and preoperative diagnosis [19].

### Definition of CR-POPF

According to the 2016 ISGPS guidelines, POPF was defined as a drain output of any measurable volume of fluid with amylase levels > 3 times the upper institutional limit of normal for serum amylase for each specific institution and association with clinically relevant development [6]. POPF was classified into three grades (A-C). Grade A implied that there was no deviation from the normal postoperative procedure and no impact on postoperative hospital stay duration. Grade B POPF required a change in the management of the expected postoperative pathway, including persistent drainage for > 3 weeks, percutaneous or endoscopic drainage, and angiographic procedure for bleeding. Grade C POPF led to organ failure, secondary operation, or subsequent POPF-related mortality. Grades B and C were defined as CR-POPF.

### Marker selection

We extracted predictive markers of CR-POPF from the previous literature, and the following 14 clinicopathological markers were used as CR-POPF predictors: age [20], sex [21], BMI [22], VFA [23], PNI [24], serum albumin level [22], parenchymal thickness [25], MPD size [26], MPD index [18], presence of diabetes mellitus [27], presence of preoperative biliary drainage [28], CONUT score [29], presence of sarcopenia [30], and preoperative diagnosis [21].

**Fig. 1:**
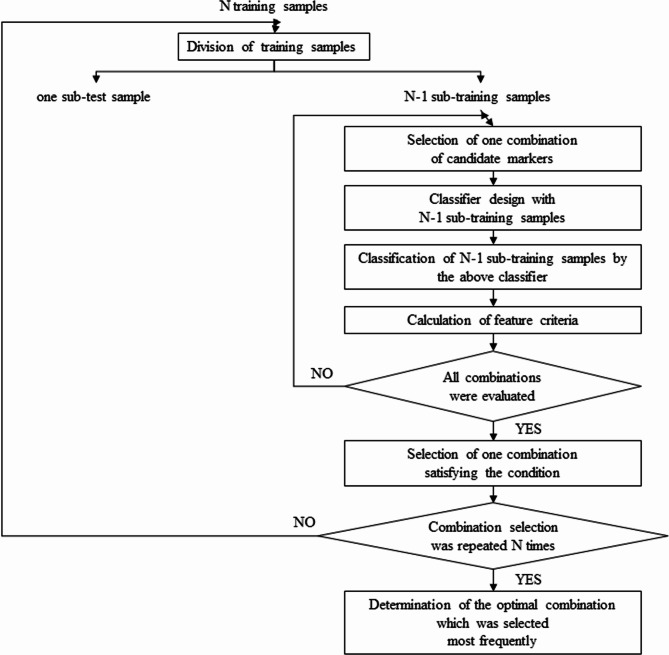
Selection of the optimal combination of markers

Among the candidate markers, a combination of two markers was selected under the conditions and examined. There were N training samples in total. Of note, marker selection was performed using only the training samples. In pattern recognition fields, markers cannot be selected based on their individual effectiveness [31]. Therefore, the combination of these markers should be carefully selected. As shown in Fig. 1, we used the leave-one-out method [32] to identify the optimal combination of markers. According to this method, one training sample was selected as a sub-test sample from N training samples, and the remaining N­1 training samples were assigned as sub-training samples. To explore marker combinations, we initially selected one combination of two markers. The discrete Bayes classifier (Additional File) was designed using N­1 sub-training samples, and the re-substitution estimate was obtained by classifying N­1 sub-training samples using the classifier. Next, feature criteria, such as sensitivity, specificity, and F1 measure, were calculated. One sub-test sample was not used for marker selection. This process was repeated until all two-marker combinations were evaluated. Among all two-marker combinations, a combination with either maximal sensitivity, subjected to a specificity ≥ 50%, or a maximal F1 measure was selected. In the leave-one-out method, the aforementioned process was repeated N times (i.e., until each training sample had been selected only once as a sub-test sample). Among the N resulting combinations, the most frequently selected combination in the leave-one-out loop was considered optimal. When the number of markers was 3, marker selection was repeated according to the same procedure. Additional details concerning the discrete Bayes classifier were provided in previous studies [13, 33, 34].

## Results

### Patient characteristics

Table 1 showed the demographic and clinicopathological characteristics of 180 patients from Yamaguchi University and 366 patients from Osaka University who underwent PD. According to the ISGPF classification, CR-POPF occurred in 60 (33%) patients of the training set and 130 (36%) patients of the validation set, with no significant difference between two groups. In-hospital mortality was 1.7% in the training set and 0.6% in the validation set, with no significant difference between the two groups. There were no significant differences in the baseline and clinical data between the training and validation sets, except that the training samples had higher preoperative albumin levels (*P* < 0.001), higher PNI (*P* < 0.001), thicker pancreatic parenchyma (*P* = 0.0007), larger MPD size (*P* = 0.023), higher frequency of samples with CONUT score ≥ 3, higher frequency of samples with pancreatic ductal adenocarcinoma (PDAC) (*P* = 0.0004), and lower frequency of preoperative radiation (*P* < 0.0001). Regarding pancreatic-enteric anastomosis, all were pancreatojejunostomy with no pancreatogastrostomy in the training set, whereas in the validation set, pancreatojejunostomy and pancreatogastrostomy were performed in 335 (91.5%) and 31 (8.5%) samples, respectively. However, there was no difference in operative time and blood loss between the training and validation sets.

**Table 1:** Demographic and clinical characteristics of the training and validation sets

	Training set N = 180 N (col%)	Validation set N = 366 N (col%)	*P-value*
CR-POPF			0.634
Present	60 (33)	130 (36)	
Absent	120 (67)	236 (64)	
Male sex	107 (59)	225 (61)	0.709
Age, median (range), years	69 (45–87)	70 (21–86)	0.891
BMI, median (range)	22.3 (15.4–33.9)	21.7 (12.5–36.2)	0.189
VFA (cm^2^)	90.0 (7.2–303.0)		
Albumin (g/dL)	4.0 (2.4-5.0)	3.8 (2.3–4.9)	< 0.001
PNI	47.7 (29.2–61.8)	45.7 (29.1–60.7)	< 0.001
Parenchymal thickness (mm)	13.0 (5.0–24.0)	11.9 (4.5–31.7)	0.0007
MPD size (mm)	4.0 (2.0–15.0)	3.95 (1.0-25.9)	0.023
MPD index	0.33 (0.11–0.92)	0.34 (0.07-1.00)	0.592
Diabetes mellitus			0.636
Yes	62 (34)	134 (37)	
No	118 (66)	232 (63)	
CONUT score			0.096
≥3	56 (31)	117 (39)	
<3	124 (69)	185 (61)	
Preoperative biliary drainage			0.579
Yes	76 (42)	145 (40)	
No	104 (58)	221 (60)	
Sarcopenia			
Yes	96 (53)		
No	84 (47)		
Preoperative diagnosis			0.0004
PDAC	84 (47)	137 (37)	
Ampullary carcinoma	29 (16)	28 (8)	
Cholangiocarcinoma	21 (12)	61 (17)	
IPMN	22 (12)	85 (23)	
Others	24 (13)	55 (15)	
Preoperative radiation			< 0.0001
Yes	5 (3)	60 (16)	
No	175 (97)	306 (84)	
Pancreatic-enteric anastomosis			< 0.001
Pancreaticojejunostomy	180 (100)	335 (91)	
Pancreaticogastrostomy	0 (0)	31 (9)	
Operation time (minutes)	524 (325–1080)	520 (285–1121)	0.292
Blood loss (mL)	590	600	0.449
	(70-7806)	(10-16070)	
In-hospital mortality	3 (1.7)	2 (0.6)	0.338

BMI, body mass index; CONUT score, controlling nutritional status score; CR-POPF, clinically relevant postoperative pancreatic fistula; IPMN, intraductal papillary mucinous neoplasm; MPD, main pancreatic duct; MPD index, MPD size/parenchymal thickness; PDAC, pancreatic ductal adenocarcinoma; PNI, prognostic nutrition index; VFA, visceral fat area.

### Assessment of optimal marker combination for the training samples

**Fig. 2:**
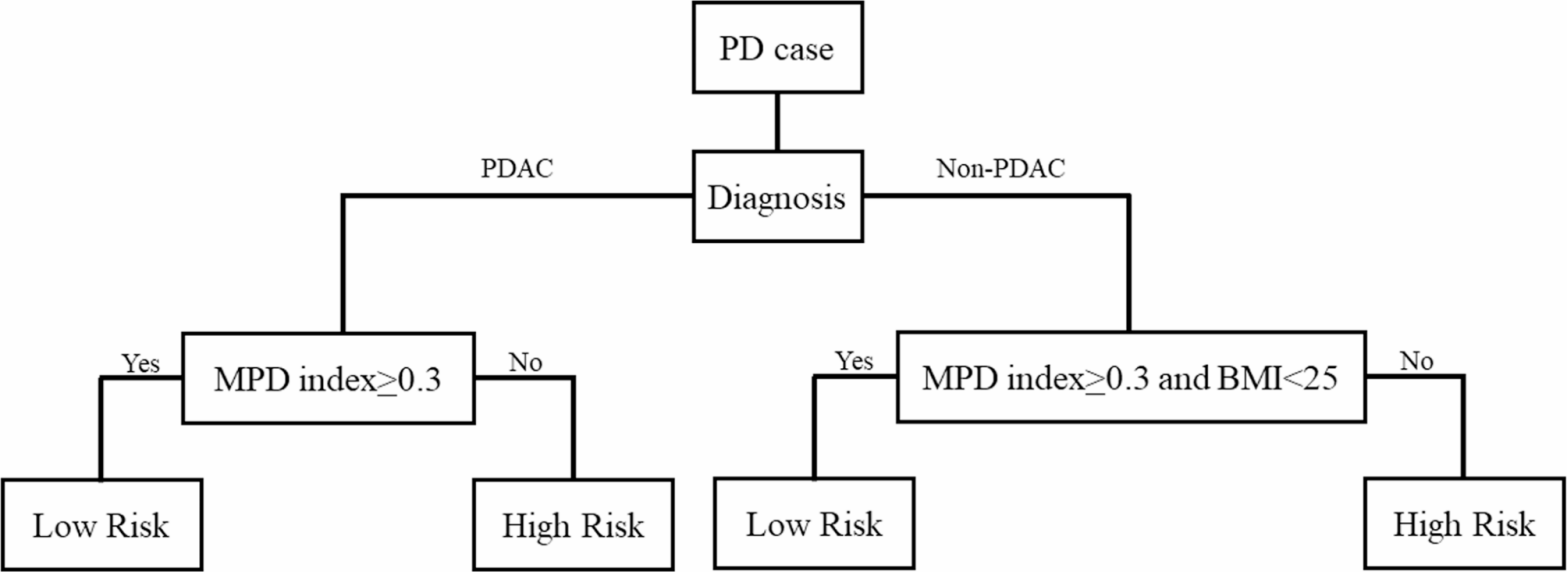
Prediction model for clinically relevant postoperative pancreatic fistula (CR-POPF).

Initially, we performed analyses on all cases. Nevertheless, we could not develop highly accurate risk models (Additional Table). It has been reported that the pancreatic exocrine function is involved in pancreatic fistulas; generally, the exocrine function is often impaired in pancreatic cancer compared with non-pancreatic cancer [35]. Therefore, we differentiated between pancreatic and non-pancreatic cancers; moreover, we performed analyses separately within each group by categorizing all cases into pancreatic cancer and non-pancreatic cancer groups. Marker selection was performed using 84 PDAC training samples from a total of 180 available training samples from Yamaguchi University. According to the leave-one-out method with 84 PDAC training samples on the discrete Bayes classifier, the optimal combination of predictive markers was determined (Fig. 1), and classification performance for the combination was then evaluated. Table 2 shows the diagnostic potential of CR-POPF using various combinations of predictive markers in the discrete Bayes classifier. For PDAC, we could differentiate CR-POPF with 87% sensitivity, 81% specificity, 50% positive predictive value, 97% negative predictive value, and F1 measure of 0.63, using only the MPD index. In contrast, the results of the two-marker combination on the discrete Bayes classifier were as follows: 93% sensitivity, 73% specificity, 44% positive predictive value, 98% negative predictive value, and F1-measure of 0.60 for the combination of MPD index and BMI; 73% sensitivity, 94% specificity, 73% positive predictive value, 94% negative predictive value, and F-measure of 0.73 for the combination of MPD index and presence of sarcopenia; 87% sensitivity, 81% specificity, 50% positive predictive value, 97% negative predictive value, and F-measure of 0.63 for the combination of MPD index and PNI; and 87% sensitivity, 81% specificity, 50% positive predictive value, 97% negative predictive value, and F-measure of 0.63 for the combination of MPD index and presence of preoperative biliary drainage. Next, marker selection was conducted for 96 non-PDAC training samples. The design of the discrete Bayes classifier with the selected combination and the evaluation of its classification performance were performed in the same manner as for PDAC samples. MPD index as a single marker resulted in 78% sensitivity, 63% specificity, 65% positive predictive value, 72% negative predictive value, and F-measure of 0.71. In contrast, we could differentiate CR-POPF with 84% sensitivity, 57% specificity, 63% positive predictive value, 80% negative predictive value, and F-measure of 0.72 using the combination of MPD index and BMI. The results of the three-marker combination on the discrete Bayes classifier were as follows: 98% sensitivity, 39% specificity, 59% positive predictive value, 96% negative predictive value, and F-measure of 0.73 for the combination of BMI, VFA, and MPD size; 84% sensitivity, 57% specificity, 63% positive predictive value, 80% negative predictive value, and F-measure of 0.72 for the combination of MPD index, age, and BMI; and 84% sensitivity, 57% specificity, 63% positive predictive value, 80% negative predictive value, and F-measure of 0.72 for the combination of MPD index, presence of sarcopenia, and BMI.　Therefore, we adopted the MPD index as a single marker for PDAC and MPD index/BMI as a two-marker combination for non-PDAC. For all samples in the training set, this prediction model showed 85% sensitivity, 71% specificity, 70% positive predictive value, and 90% negative predictive value. Based on these results, we developed a prediction model as shown in Fig. 2.

**Table 2:** Classification results for the training set

Predictive Marker		Sensitivity (%)	Specificity (%)	PPV (%)	NPV (%)
**PDAC training samples**					
	MPD index (cut-off 0.3)	87	81	50	97
	MPD index (cut-off 0.3), sarcopenia (+/-)	73	94	73	94
	MPD index (cut-off 0.3), PNI (cut-off 45)	87	81	50	97
	MPD index (cut-off 0.3), preoperative biliary drainage (+/-)	87	81	50	97
**Non-PDAC training samples**					
	MPD index (cut-off 0.3)	78	63	65	72
	MPD index (cut-off 0.3), BMI (cut-off 25)	84	57	63	80
	BMI (cut-off 0.3), VFA (cut-off 100), MPD size (cut-off 3)	98	39	59	96
	MPD index (cut-off 0.3), age (cut-off 65), BMI (cut-off 25)	84	57	63	80
	MPD index (cut-off 0.3), sarcopenia (+/-), BMI (cut-off 25)	84	57	63	80

BMI, body mass index; MPD, main pancreatic duct; MPD index, MPD size/parenchymal thickness; NPV, negative predictive value; PDAC, pancreatic ductal adenocarcinoma; PNI, prognostic nutritional index; PPV, positive predictive value; VFA, visceral fat area.

### Validation of prediction model

We used a validation set of 366 patients from Osaka University, which was independent of the training set. The results are shown in Tables 3 and 4. For 130 PDAC test samples in the validation set, the prediction model using only the MPD index had 93% sensitivity, 87% specificity, 47% positive predictive value, and 99% negative predictive value. For 236 non-PDAC test samples in the validation set, the prediction model using the two-marker combination of MPD index and BMI showed 85% sensitivity, 53% specificity, 64% positive predictive value, and 78% negative predictive value. For all test samples in the validation set, this prediction model showed 86% sensitivity, 71% specificity, 62% positive predictive value, and 90% negative predictive value.

**Table 3:** Classification results for the validation set

Predictive Marker	Sensitivity (%)	Specificity (%)	PPV (%)	NPV (%)
**PDAC test samples**				
MPD index (cut-off 0.3)	93	87	47	99
**Non-PDAC test samples**				
MPD index (cut-off 0.3), BMI (cut-off 25)	85	53	64	78

BMI, body mass index; MPD, main pancreatic duct; MPD index, MPD size/parenchymal thickness; NPV, negative predictive value; PDAC, pancreatic ductal adenocarcinoma; PPV, positive predictive value.

**Table 4:** Confusion matrix of the prediction results on validation set for CR-POPF

PDAC cases			
		Actual	
		CR-POPF (+)	CR-POPF (-)
Predicted	CR-POPF (+)	14	16
	CR-POPF (-)	1	104
	Total	15	120
**Non-PDAC cases**			
		**Actual**	
		**CR-POPF (+)**	**CR-POPF (-)**
Predicted	CR-POPF (+)	98	53
	CR-POPF (-)	17	61
	Total	115	114

CR-POPF, clinically relevant postoperative pancreatic fistula; PDAC, pancreatic ductal adenocarcinoma

## Discussion

This study established a novel prediction model of CR-POPF after PD by using a discrete Bayes classifier with only preoperative parameters. As a result, the prediction model was constructed using the preoperative diagnosis of disease, MPD index (ratio of pancreatic duct diameter/pancreatic parenchymal diameter) [18], and BMI to stratify the training samples into high- and low-risk groups. This prediction model was as accurate in the validation set as in the training set. The ISGPS recommends the use of an external pancreatic duct stent and somatostatin analogs for high-risk samples after PD [8]. Recent reports also suggested that pancreatojejunostomy was better than pancreatogastrostomy for high-risk samples [7]. However, there are several advantages to predicting the risk of CR-POPF not only in terms of the choice of surgical technique and perioperative management but also in other aspects. For example, based on the risk of CR-POPF, preoperative informed consent can be provided more cautiously. Specifically, in frail and vulnerable patients with benign or borderline tumors, or with uncertain pathological behavior, the preoperative risk prediction may be helpful when deciding whether to pursue conservative treatment or surgical intervention. To implement such a policy, it is important to identify high-risk samples preoperatively. Several useful single risk markers for pancreatic fistula were previously reported. However, POPF is confounded by multiple markers, and its accurate prediction based on single risk markers is difficult. Therefore, it is important to create a more accurate predictive model by combining multiple risk markers with documented usefulness. Several risk models for POPF using multiple markers have been reported. Many previous reports used intraoperative or postoperative parameters, such as intraoperative blood loss or drain findings, which make it difficult to preoperatively schedule the details of the surgical procedure or prepare the medications to be used [36]. Nevertheless, in recent years, there have been several reports of predictive models using only preoperative parameters [37–39]. These models combined independent risk markers extracted from univariate and multivariate analyses. However, the combination of multiple markers extracted in this manner is not always optimal. We differentiated CR-POPF by using an original discrete Bayes classifier [13], which is unique and can handle both non-numerical and numerical data on the basis of Bayes’ decision theory using posterior probability. The leave-one-out method was adopted in the model design to explore the optimal combination of markers and evaluate the distinguishability. Using this estimation, we can eliminate ambiguity in the determination of CR-POPF and obtain an objective probability distribution.

Our risk model first classified PDAC and non-PDAC during the development of our risk model. As shown in the Additional Table, the classification performance for the training set with stratified PDAC vs. non-PDAC samples was superior to that of the training set with two groups combined. This finding might result from a difference in the mechanism by which pancreatic fistulas develop between PDAC and non-PDAC samples.

As previously reported, many PDAC samples have increased physical strength due to fibrosis caused by pancreatitis associated with pancreatic duct obstruction, resulting in “hard pancreas” [40]. Additionally, in PDAC samples, the distal MPD is dilated and easily sutured during surgery. The passage of pancreatic juice through the anastomosis might be smooth. In PDAC samples, the decline of exocrine function may lead to a decreased frequency of CR-POPF [41]. In contrast, most non-PDAC samples are “soft pancreas” with little MPD dilatation, little fibrosis of parenchyma, and preserved exocrine function. Therefore, PDAC or non-PDAC may be major predictors of a soft or hard pancreas, respectively. In PDAC samples, the classification performance of a single marker (MPD index) was optimal without requirement for combination with other markers. As mentioned above, most pancreatic samples are hard pancreas. However, not all PDAC samples are hard pancreas, and lesions in the uncinate process of the pancreas or groove region may not be hard pancreas because there is no MPD obstruction. As a result, the MPD index will be low in such samples because the pancreatic parenchyma does not undergo atrophy without MPD dilatation. Therefore, a lower MPD index may be a predictor of soft pancreas, which is susceptible to CR-POPF, in PDAC samples, and is clinically reasonable.

In contrast, for non-PDAC samples, the two-marker combination was superior to a single marker, but a three-marker combination was not necessary. As described above, most non-PDAC samples were soft pancreas with a thin MPD and non-atrophied parenchyma, resulting in a lower MPD index. These samples may be at a high risk for CR-POPF because of the fragile pancreas and preserved pancreatic exocrine function. However, even in non-PDAC samples, when the MPD is obstructed by an invading lesion, the MPD index is higher because of MPD dilatation and fibrotic atrophy of the pancreatic parenchyma, which results in a hard pancreas. Such samples are less likely to develop pancreatic fistulas because they have the same mechanism as PDAC samples. However, non-PDAC samples, such as some samples of intraductal papillary mucinous neoplasm, may sometimes have a dilated MPD and high MPD index but are soft pancreas without obstructive pancreatitis and fibrosis [42]. In such samples, a high MPD index can be a false negative for CR-POPF risk. Therefore, BMI may be a complementary risk factor in such samples. BMI was reportedly associated with soft-textured fatty pancreas and CR-POPF. The physical fragility of the fatty pancreas may be the cause of CR-POPF [10].

Although significant differences could not be examined because this was a one-time result, the performance of our predictive model for the test samples in the validation set was similar to that of the training sample. While the performance of the predictive model generally degrades with test samples as compared to training samples, no degradation was observed because our prediction model was highly accurate. In particular, the prediction accuracy was maintained despite some background differences between the training and validation sets. Notably, the training set included only pancreatojejunostomy, while the validation set included both pancreatojejunostomy and pancreatogastrostomy. However, the performance of our prediction model for the validation set was not degraded.

POPF risk prediction can be influenced by a variety of factors, including the surgeon’s level of training and differences in management at the facility. In this study, the training and validation samples were obtained at different facilities, and the validation sample had a larger number of cases than the training sample; nonetheless, the validation sample had the same prediction accuracy as the training sample. The validation sample usually has a lower prediction accuracy than the training sample, and therefore this prediction system may be reproducible. For example, in this study, the cutoff for BMI was set at 25 kg/m^2^, which corresponds to the standard threshold for obesity as defined by the Japanese Society for the Study of Obesity [43]. However, this threshold may be different in other countries. Since our study sample was limited to Japanese participants, more research is required to verify our findings in individuals from other countries.

This study had several limitations. First, the data were collected retrospectively. There were variations in the observation points of the data (e.g., timing of blood biochemistry and CT scans). Additionally, owing to insufficient pathological samples of the remaining pancreatic transection, we were unable to verify the pathology of our hypotheses. Second, the frequency of CR-POPF at these two facilities is higher than that reported in recent years [6]. This is because these two facilities used to be cautious in dealing with POPF and actively used octreotide and replaced drain based on strict criteria. This may have led to overtreatment for some cases. With the current improvement in treatment practices, the use of such procedures is gradually decreasing. Hence, the in-hospital mortality rate was lower in our study than that previously reported [44]. Recent studies have reported the efficacy of drain replacement and octreotide administration in patients with CR-POPF before serious complications develop [45, 46]. Therefore, it would be necessary to analyze the countermeasure for POPF; however, this was a retrospective study, and thus we could not perform the analysis due to missing data. Finally, because the results were obtained at only two facilities, there was some concern that the MPD index measurement results might vary if the results were verified in a larger number of facilities. Therefore, our predictive model should be validated in future prospective studies using a larger number of centers. Our next goal is to conduct a larger prospective study and identify high-risk patients for whom CR-POPF should be managed preoperatively and intraoperatively.

## Conclusions

We developed a novel prediction model for pancreatic fistulas after　PD using only preoperative markers. For CR-POPF assessment, the MPD index will be useful in PDAC samples, while the MPD index/BMI combination will be useful in non-PDAC samples.

### Electronic supplementary material

Below is the link to the electronic supplementary material.

Supplementary Material 1

Supplementary Material 2

## Data Availability

The datasets used and/or analyzed during the current study are available from the corresponding author (HN). on reasonable request.

## List of abbreviations

BMI: body mass index
CONUT: controlling nutritional status
CR-POPF: clinically relevant postoperative pancreatic fistula
CT: computed tomography
ISGPS: International Study Group of Pancreatic Surgery
MPD: main pancreatic duct
PD: pancreaticoduodenectomy
PDAC: pancreatic ductal adenocarcinoma
PNI: prognostic nutritional index
VFA: visceral fat area

## References

[1] Panni RZ, Panni UY, Liu J, Williams GA, Fields RC, Sanford DE, Hawkins WG, Hammill CW (2021). Re-defining a high volume center for pancreaticoduodenectomy. HPB.

[2] Fukami Y, Saito T, Osawa T, Komatsu S, Sano T (2022). Blumgart anastomosis with polyglycolic acid felt reduces the incidence of pancreatic fistula after pancreaticoduodenectomy: a propensity score analysis. Annals of Gastroenterological Surgery.

[3] Mizushima T, Yamamoto H, Marubashi S, Kamiya K, Wakabayashi G, Miyata H, Seto Y, Doki Y, Mori M (2018). Validity and significance of 30-day mortality rate as a quality indicator for gastrointestinal cancer surgeries. Ann Gastroenterol Surg.

[4] Yeo CJ, Cameron JL, Sohn TA, Lillemoe KD, Pitt HA, Talamini MA, Hruban RH, Ord SE, Sauter PK, Coleman J (1997). Six hundred fifty consecutive pancreaticoduodenectomies in the 1990s: pathology, complications, and outcomes. Ann Surg.

[5] Muscari F, Suc B, Kirzin S, Hay J-M, Fourtanier G, Fingerhut A, Sastre B, Chipponi J, Fagniez P-L, Radovanovic A (2006). Risk factors for mortality and intra-abdominal complications after pancreatoduodenectomy: multivariate analysis in 300 patients. Surgery.

[6] Bassi C, Marchegiani G, Dervenis C, Sarr M, Hilal MA, Adham M, Allen P, Andersson R, Asbun HJ, Besselink MG (2017). The 2016 update of the International Study Group (ISGPS) definition and grading of postoperative pancreatic fistula: 11 years after. Surgery.

[7] Ecker BL, McMillan MT, Asbun HJ, Ball CG, Bassi C, Beane JD, Behrman SW, Berger AC, Dickson EJ, Bloomston M (2018). Characterization and optimal management of high-risk pancreatic anastomoses during pancreatoduodenectomy. Ann Surg.

[8] Shrikhande SV, Sivasanker M, Vollmer CM, Friess H, Besselink MG, Fingerhut A, Yeo CJ, Fernandez-delCastillo C, Dervenis C, Halloran C (2017). Pancreatic anastomosis after pancreatoduodenectomy: a position statement by the International Study Group of pancreatic surgery (ISGPS). Surgery.

[9] Rosso E, Casnedi S, Pessaux P, Oussoultzoglou E, Panaro F, Mahfud M, Jaeck D, Bachellier P (2009). The role of fatty pancreas and of BMI in the occurrence of pancreatic fistula after pancreaticoduodenectomy. J Gastrointest Surg.

[10] Gaujoux S, Cortes A, Couvelard A, Noullet S, Clavel L, Rebours V, Lévy P, Sauvanet A, Ruszniewski P, Belghiti J (2010). Fatty pancreas and increased body mass index are risk factors of pancreatic fistula after pancreaticoduodenectomy. Surgery.

[11] Fukami Y, Saito T, Osawa T, Hanazawa T, Kurahashi T, Kurahashi S, Matsumura T, Komatsu S, Kaneko K, Sano T (2021). Which is the best predictor of clinically relevant pancreatic fistula after pancreatectomy: drain fluid concentration or total amount of amylase?. Annals of Gastroenterological Surgery.

[12] Mungroop TH, Van Rijssen LB, Van Klaveren D, Smits FJ, Van Woerden V, Linnemann RJ, De Pastena M, Klompmaker S, Marchegiani G, Ecker BL (2019). Alternative fistula risk score for pancreatoduodenectomy (a-FRS): design and international external validation. Ann Surg.

[13] Ogihara H, Iizuka N, Hamamoto Y. Prediction of early recurrence of liver cancer by a novel discrete bayes decision rule for personalized medicine. *BioMed research international* 2016, 2016. 10.1155/2016/8567479 PMC5075355 27800494

[14] Hasegawa K, Kokudo N, Sano K, Seyama Y, Aoki T, Ikeda M, Hashimoto T, Beck Y, Imamura H, Sugawara Y (2008). Two-stage pancreatojejunostomy in pancreaticoduodenectomy: a retrospective analysis of short-term results. Am J Surg.

[15] Onodera T, Goseki N, Kosaki G (1984). Prognostic nutritional index in gastrointestinal surgery of malnourished cancer patients. Nihon Geka Gakkai Zasshi.

[16] De Ulíbarri JI, González-Madroño A, de Villar NG, González P, González B, Mancha A, Rodríguez F, Fernández G (2005). CONUT: a tool for controlling nutritional status. First validation in a hospital population. Nutr Hosp.

[17] Shimizu A, Tani M, Kawai M, Hirono S, Miyazawa M, Uchiyama K, Yamaue H (2011). Influence of visceral obesity for postoperative pulmonary complications after pancreaticoduodenectomy. J Gastrointest Surg.

[18] Akamatsu N, Sugawara Y, Komagome M, Shin N, Cho N, Ishida T, Ozawa F, Hashimoto D (2010). Risk factors for postoperative pancreatic fistula after pancreaticoduodenectomy: the significance of the ratio of the main pancreatic duct to the pancreas body as a predictor of leakage. J Hepato-Biliary-Pancreat Sci.

[19] Nishida Y, Kato Y, Kudo M, Aizawa H, Okubo S, Takahashi D, Nakayama Y, Kitaguchi K, Gotohda N, Takahashi S (2016). Preoperative sarcopenia strongly influences the risk of postoperative pancreatic fistula formation after pancreaticoduodenectomy. J Gastrointest Surg.

[20] Wellner UF, Kayser G, Lapshyn H, Sick O, Makowiec F, Höppner J, Hopt UT, Keck T (2010). A simple scoring system based on clinical factors related to pancreatic texture predicts postoperative pancreatic fistula preoperatively. Hpb.

[21] Yamamoto Y, Sakamoto Y, Nara S, Esaki M, Shimada K, Kosuge T (2011). A preoperative predictive scoring system for postoperative pancreatic fistula after pancreaticoduodenectomy. World J Surg.

[22] Yu L, Huang Q, Xie F, Lin X, Liu C (2014). Risk factors of postoperative complications of pancreatoduodenectomy. Hepatogastroenterology.

[23] Tranchart H, Gaujoux S, Rebours V, Vullierme M-P, Dokmak S, Levy P, Couvelard A, Belghiti J, Sauvanet A (2012). Preoperative CT scan helps to predict the occurrence of severe pancreatic fistula after pancreaticoduodenectomy. Ann Surg.

[24] Kanda M, Fujii T, Kodera Y, Nagai S, Takeda S, Nakao A (2011). Nutritional predictors of postoperative outcome in pancreatic cancer. J Br Surg.

[25] Sugimoto M, Takahashi S, Kojima M, Kobayashi T, Gotohda N, Konishi M (2017). In patients with a soft pancreas, a thick parenchyma, a small duct, and fatty infiltration are significant risks for pancreatic fistula after pancreaticoduodenectomy. J Gastrointest Surg.

[26] Wada K, Traverso LW (2006). Pancreatic anastomotic leak after the Whipple procedure is reduced using the surgical microscope. Surgery.

[27] Lin JW, Cameron JL, Yeo CJ, Riall TS, Lillemoe KD (2004). Risk factors and outcomes in postpancreaticoduodenectomy pancreaticocutaneous fistula. J Gastrointest Surg.

[28] Fujii T, Yamada S, Suenaga M, Kanda M, Takami H, Sugimoto H, Nomoto S, Nakao A, Kodera Y (2015). Preoperative internal biliary drainage increases the risk of bile juice infection and pancreatic fistula after pancreatoduodenectomy: a prospective observational study. Pancreas.

[29] Utsumi M, Aoki H, Nagahisa S, Nishimura S, Une Y, Kimura Y, Watanabe M, Taniguchi F, Arata T, Katsuda K (2020). Preoperative predictive factors of pancreatic fistula after pancreaticoduodenectomy: usefulness of the CONUT score. Annals of Surgical Treatment and Research.

[30] Nishida Y, Kato Y, Kudo M, Aizawa H, Okubo S, Takahashi D, Nakayama Y, Kitaguchi K, Gotohda N, Takahashi S (2016). Preoperative sarcopenia strongly influences the risk of postoperative pancreatic fistula formation after pancreaticoduodenectomy. J Gastrointest Surg.

[31] Jain AK, Duin RPW, Mao J (2000). Statistical pattern recognition: a review. IEEE Trans Pattern Anal Mach Intell.

[32] Lachenbruch PA, Mickey MR (1968). Estimation of error rates in discriminant analysis. Technometrics.

[33] Goto A, Nishikawa J, Hideura E, Ogawa R, Nagao M, Sasaki S, Kawasato R, Hashimoto S, Okamoto T, Ogihara H (2017). Lymph node metastasis can be determined by just tumor depth and lymphovascular invasion in early gastric cancer patients after endoscopic submucosal dissection. Eur J Gastroenterol Hepatol.

[34] Nakagami Y, Hazama S, Suzuki N, Yoshida S, Tomochika S, Matsui H, Shindo Y, Tokumitsu Y, Matsukuma S, Watanabe Y. CD4 and FOXP3 as predictive markers for the recurrence of T3/T4a stage II colorectal cancer: applying a novel discrete Bayes decision rule. 2022. 10.1186/s12885-022-10181-7 PMC9578193 36253752

[35] Callery MP, Pratt WB, Kent TS, Chaikof EL, Vollmer CM (2013). A prospectively validated clinical risk score accurately predicts pancreatic fistula after pancreatoduodenectomy. J Am Coll Surg.

[36] Li Y, Zhou F, Zhu D-M, Zhang Z-X, Yang J, Yao J, Wei Y-J, Xu Y-L, Li D-C, Zhou J (2019). Novel risk scoring system for prediction of pancreatic fistula after pancreaticoduodenectomy. World J Gastroenterol.

[37] Yamamoto Y, Sakamoto Y, Nara S, Esaki M, Shimada K, Kosuge T (2011). A preoperative predictive scoring system for postoperative pancreatic fistula after pancreaticoduodenectomy. World J Surg.

[38] Perri G, Marchegiani G, Partelli S, Crippa S, Bianchi B, Cinelli L, Esposito A, Pecorelli N, Falconi M, Bassi C (2021). Preoperative risk stratification of postoperative pancreatic fistula: a risk-tree predictive model for pancreatoduodenectomy. Surgery.

[39] van Dongen JC, van Dam JL, Besselink MG, Bonsing BA, Bosscha K, Busch OR, van Dam RM, Festen S, van der Harst E, de Hingh IH. Fistula Risk score for auditing pancreatoduodenectomy: the auditing FRS. Ann Surg. 2022. 10.1097. 10.1097/SLA.0000000000005532 35837978

[40] Eshmuminov D, Schneider MA, Tschuor C, Raptis DA, Kambakamba P, Muller X, Lesurtel M, Clavien P-A (2018). Systematic review and meta-analysis of postoperative pancreatic fistula rates using the updated 2016 International Study Group Pancreatic Fistula definition in patients undergoing pancreatic resection with soft and hard pancreatic texture. Hpb.

[41] Adachi E, Harimoto N, Yamashita Y-i, Sakaguchi Y, Toh Y, Okamura T, Nishiyama K, Saeki H, Uchiyama H, Morita M. Pancreatic leakage test in pancreaticoduodenectomy: relation to degree of pancreatic fibrosis, pancreatic amylase level and pancreatic fistula. 2013. 24693676

[42] Khoury RE, Kabir C, Maker VK, Banulescu M, Wasserman M, Maker AV (2018). What is the incidence of malignancy in resected intraductal papillary mucinous neoplasms? An analysis of over 100 US institutions in a single year. Ann Surg Oncol.

[43] Kanazawa M, Yoshiike N, Osaka T, Numba Y, Zimmet P, Inoue S (2002). Criteria and classification of obesity in Japan and Asia-Oceania. Asia Pac J Clin Nutr.

[44] Merath K, Mehta R, Tsilimigras DI, Farooq A, Sahara K, Paredes AZ, Wu L, Ejaz A, Pawlik TM (2020). In-hospital mortality following pancreatoduodenectomy: a comprehensive analysis. J Gastrointest Surg.

[45] Adachi T, Ono S, Matsushima H, Soyama A, Hidaka M, Takatsuki M, Eguchi S (2019). Efficacy of triple-drug therapy to prevent pancreatic fistulas in patients with high drain amylase levels after pancreaticoduodenectomy. J Surg Res.

[46] Zhao N, Cui J, Yang Z, Xiong J, Wu H, Wang C, Peng T. Natural history and therapeutic strategies of post-pancreatoduodenectomy abdominal fluid collections: Ten-year experience in a single institution. *Medicine* 2019, 98(22). 10.1097/MD.0000000000015792 PMC6708627 31145305

