# Supplementary material for: A novel prediction model of pancreatic fistula after pancreaticoduodenectomy using only preoperative markers
Source: BMC Surg. 2023 Oct 12;23:310. doi: 10.1186/s12893-023-02213-1 (PMC10571374; doi:10.1186/s12893-023-02213-1)
Supplement: Supplementary file 1 — Supplementary Material 1 [file 12893_2023_2213_MOESM1_ESM.docx]

| **Additional table.** Classification results for the training set without stratifying PDAC and non-PDAC. | | | | | |
| --- | --- | --- | --- | --- | --- |
| Marker | | Sensitivity  (%) | Specificity  (%) | PPV  (%) | NPV  (%) |
|  | MPD index<0.3 | 80 | 73 | 60 | 88 |
|  | MPD index<0.3 and Sarcopenia- | 80 | 73 | 60 | 88 |
|  | MPD index<3 and BMII>25 | 83 | 63 | 53 | 88 |
|  | MPD index<0.3 and BMI>25 and Male | 77 | 78 | 64 | 87 |
|  | MPD index<0.3 and BMI>25 and Age<65 | 87 | 67 | 56 | 91 |
|  | MPD index<0.3 and BMI>25 and preoperative biliary drainage+ | 87 | 67 | 56 | 91 |
|  | MPD index<0.3 and BMI>25 and sarcopenia+ | 87 | 67 | 56 | 91 |
| MPD, main pancreatic duct; MPD index, MPD size/Parenchymal thickness; BMI, body mass index;  PPV, positive predictive value; NPV, negative predictive value | | | | | |

**LEGENDS**

As shown in the Supplementary Table, the classification performance for the training set with stratified PDAC vs. non-PDAC samples was superior to that of the training set with two groups combined. This finding might result from a difference in the mechanism by which pancreatic fistulas develop between PDAC and non-PDAC samples.
