# Supplementary material for: A novel prediction model of pancreatic fistula after pancreaticoduodenectomy using only preoperative markers
Source: BMC Surg. 2023 Oct 12;23:310. doi: 10.1186/s12893-023-02213-1 (PMC10571374; doi:10.1186/s12893-023-02213-1)

**Additional File**

**Discrete Bayes Classifier**

The discrete Bayes classifier is characterized by the fact that it can handle discrete data. $x_{1}$ and$x_{2}$are given as two markers. The range of each marker is exclusively divided into divisions.

Suppose that$x_{1} has 2 divisions and x_{2} has 3 divisions.$ Moreover, the discretized data of a patient belong to the first division $x_{1(1)}$ in marker $x_{1}$and the third division $x_{2\left( 3 \right)}$in marker $x_{2}.$That is, $\mathbf{x}$ = ($x_{1\left( 1 \right)}, x_{2\left( 3 \right)}$). Then, $P\left( x_{1\left( 1 \right)} | \omega_{1} \right)$ and $P\left( x_{2\left( 3 \right)} | \omega_{1} \right)$ for class $\omega_{1}$ are defined as follows:

$$P\left( x_{1\left( 1 \right)} | \omega_{1} \right)=\frac{n_{1(1)}^{1}}{n_{1(1)}^{1}+n_{2(3)}^{1}}$$

$$\mathrm{and}$$

$$P\left( x_{2\left( 3 \right)} | \omega_{1} \right)=\frac{n_{2(3)}^{1}}{n_{1(1)}^{1}+n_{2(3)}^{1}}$$

where $n_{1(1)}^{1}$ denotes the number of training samples ${for \omega}_{1}$ belonging to the division $x_{1(1)}$and $n_{2(3)}^{1}$ denotes the number of the training samples${for \omega}_{1}$ belonging to the division $x_{2\left( 3 \right)}.$ Then, the class-conditional probability $P\left( x_{1\left( 1 \right)},x_{2\left( 3 \right)} | \omega_{1} \right)$ for $\omega_{1}$is given by

$$P\left( x_{1\left( 1 \right)},x_{2\left( 3 \right)} | \omega_{1} \right)=P\left( x_{1\left( 1 \right)} | \omega_{1} \right)P\left( x_{2\left( 3 \right)} | \omega_{1} \right)$$

Using $n_{1(1)}^{2}$ and $n_{2\left( 3 \right)}^{2}\mathrm{for}\omega_{2}$, we similarly get

$$P\left( x_{1\left( 1 \right)} | \omega_{2} \right)=\frac{n_{1(1)}^{2}}{n_{1(1)}^{2}+n_{2(3)}^{2}}$$

$$\mathrm{and}$$

$$P\left( x_{2\left( 3 \right)} | \omega_{2} \right)=\frac{n_{2(3)}^{2}}{n_{1(1)}^{2}+n_{2(3)}^{2}}$$

$P\left( x_{1\left( 1 \right)},x_{2\left( 2 \right)} | \omega_{2} \right)$ for $\omega_{2}$ is also given by

$$P\left( x_{1\left( 1 \right)},x_{2\left( 3 \right)} | \omega_{2} \right)=P\left( x_{1\left( 1 \right)} | \omega_{2} \right)P\left( x_{2\left( 3 \right)} | \omega_{2} \right)$$

The a posteriori probabilities of classes $\omega_{i}$ are given as follows:

$$P\left( \omega_{i} \right| \mathbf{x} )=\frac{P\left( x_{1\left( 1 \right)},x_{2\left( 3 \right)} | \omega_{i} \right)}{P\left( x_{1\left( 1 \right)},x_{2\left( 3 \right)} | \omega_{1} \right)+P\left( x_{1\left( 1 \right)},x_{2\left( 3 \right)} | \omega_{2} \right)}$$

In this study, we assumed that in the *a priori* probability,$P(\omega_{i})$, $P(\omega_{1})$=$P(\omega_{2})$. Then, the patient was classified into the class in which the a posteriori probability was the maximum.

We assume that the data of a test sample belong to the second division $x_{1(2)}$ , i.e., $L$ in the MPD index $x_{1}$ and the first division $x_{2(1)}$ , i.e., $H$ in the BMI $x_{2}$. Using the partition table, $P\left( x_{1(2)} | Grades B and C \right)$, $P\left( x_{2(1)} | Grades B and C \right)$ and $P\left( x_{1(2)}, x_{2(1)} | Grades B and C \right)$ are given by

$$P\left( x_{1(2)} | Grades B and C \right)= 0.673$$

$$P\left( x_{2(1)} | Grades B and C \right)= 0.327$$

$$P\left( x_{1(2)}, x_{2(1)} | Grades B and C \right)=0.220$$

For Grade A, we similarly get

$$P\left( x_{1(2)} | Grade A \right)=0.731$$

$$P\left( x_{2(1)} | Grade A \right)= 0.269$$

$$P\left( x_{1(2)}, x_{2(1)} | Grade A \right)=0.197$$

Then, the a posteriori probabilities of two classes are given as follows:

$$P\left( Grades B and C | x_{1(2)}, x_{2(1)} \right)= 0.528$$

$$P\left( Grade A | x_{1(2)}, x_{2(1)} \right)=0.472$$

According to the Bayes decision rule, the test sample is classified into Grades B and C.


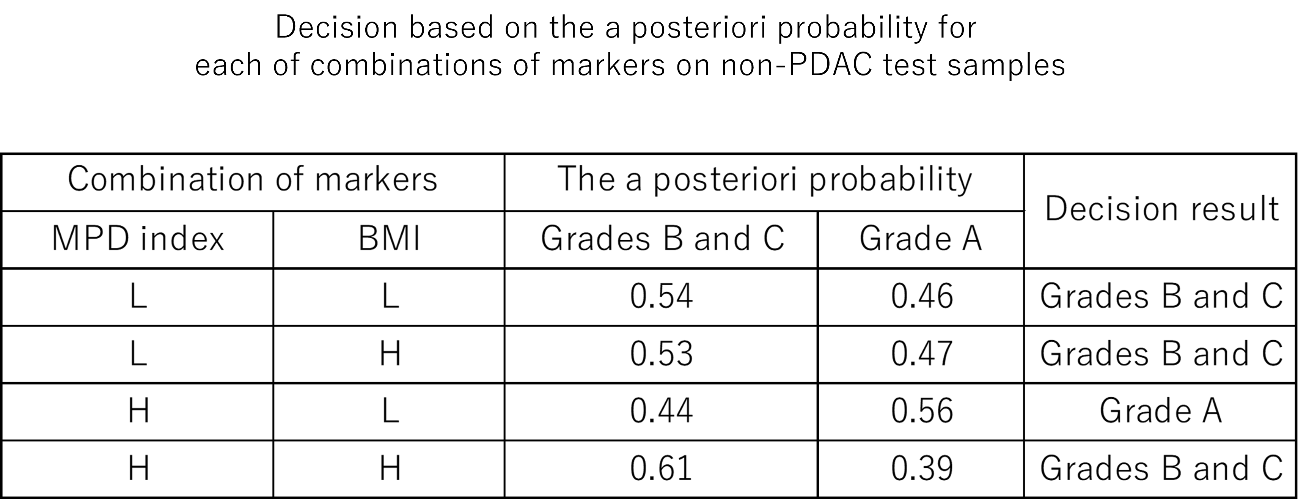

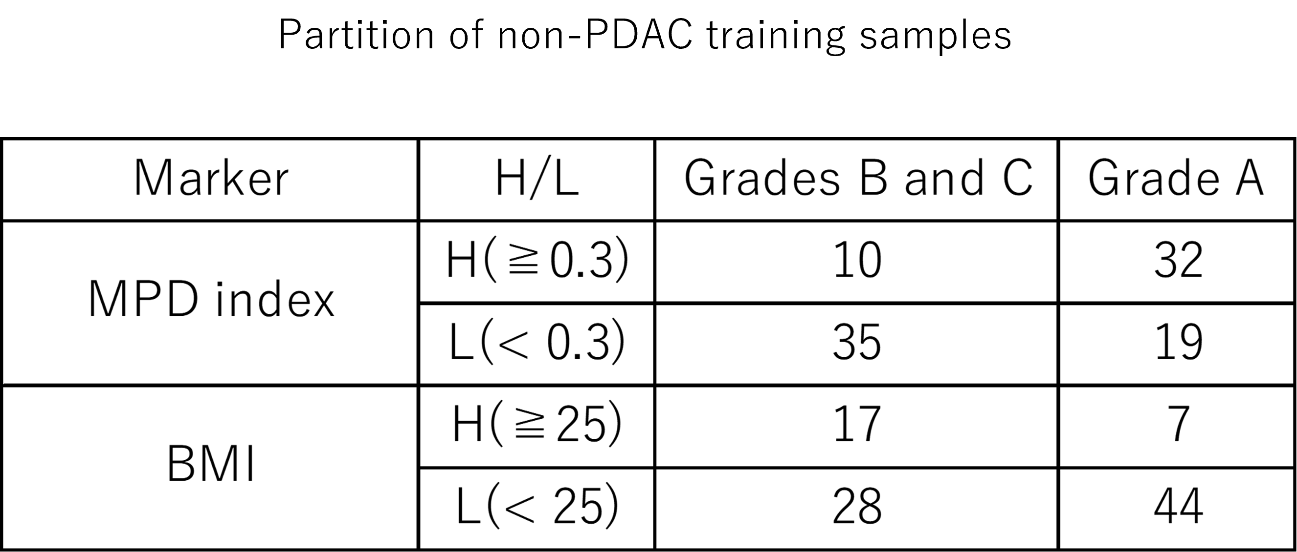

Supplement: Supplementary file 2 — Supplementary Material 2 [file 12893_2023_2213_MOESM2_ESM.docx]
